# 9β-Hy­droxy-6,9-dimethyl-3-methyl­ene-3a,4,8,9,9a,9b-hexa­hydro­azuleno[4,5-*b*]furan-2(3*H*)-one

**DOI:** 10.1107/S1600536812000165

**Published:** 2012-01-14

**Authors:** Mohamed Moumou, Ahmed Benharref, Jean Claude Daran, Fouad Mellouki, Moha Berraho

**Affiliations:** a Laboratoire de Chimie Biomoléculaire, Substances Naturelles et Réactivité, URAC 16, Faculté des Sciences Semlalia, BP 2390, Bd My Abdellah, 40000 Marrakech, Morocco; b Laboratoire de Chimie de Coordination, 205 route de Narbonne, 31077 Toulouse Cedex 04, France; c Laboratoire de Chimie Bioorganique et Analytique, URAC 22, BP 146, FSTM, Universite’ Hassan II, Mohammedia-Casablanca 20810 Mohammedia, Morocco

## Abstract

The title compound, C_15_H_18_O_3_, was synthesized from 9α-hy­droxy­parthenolide (9α-hy­droxy-4,8-dimethyl-12-methylen-3,14-dioxa-tricyclo­[9.3.0.0^2,4^]tetra­dec-7-en-13-one), which was isolated from the chloro­form extract of the aerial parts of *Anvillea radiata*. The seven-membered ring of the title compound shows a chair conformation, while the five-membered rings exibit different conformations, *viz* a twisted one for the lactone ring and an envelope conformation for the other five-membered ring with the C atom closest to the hydroxy group forming the flap. In the crystal, O—H⋯O hydrogen bonds connect mol­ecules into dimers that are inter­connected by C—H⋯O inter­actions, producing supramolecular chains along the *b* axis.

## Related literature

For background to the medicinal uses of the plant *Anvillea radiata*, see: Abdel Sattar *et al.* (1996 ▶); Bellakhdar (1997 ▶); El Hassany *et al.* (2004 ▶); Qureshi *et al.* (1990 ▶). For the reactivity of this sesquiterpene, see: El Haib *et al.* (2011 ▶) For ring puckering parameters, see: Cremer & Pople (1975 ▶).
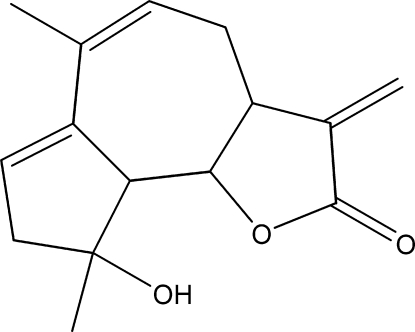


## Experimental

### 

#### Crystal data

C_15_H_18_O_3_
*M* *_r_* = 246.29
Monoclinic, 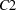

*a* = 15.6732 (9) Å
*b* = 7.4208 (4) Å
*c* = 11.0544 (6) Å
β = 103.169 (6)°
*V* = 1251.90 (12) Å^3^
*Z* = 4
Mo *K*α radiation
μ = 0.09 mm^−1^
*T* = 180 K
0.42 × 0.19 × 0.12 mm

#### Data collection

Agilent Xcalibur Eos Gemini Ultra diffractometer
Absorption correction: multi-scan (*CrysAlis PRO*; Agilent, 2010 ▶) *T*_min_ = 0.631, *T*_max_ = 1.000
13492 measured reflections
1378 independent reflections
1313 reflections with *I* > 2σ(*I*)
*R*_int_ = 0.029

#### Refinement

*R*[*F* ^2^ > 2σ(*F* ^2^)] = 0.030
*wR*(*F* ^2^) = 0.080
*S* = 1.05
1378 reflections
166 parameters
1 restraint
H-atom parameters constrained
Δρ_max_ = 0.19 e Å^−3^
Δρ_min_ = −0.15 e Å^−3^

### 

Data collection: *CrysAlis PRO* (Agilent, 2010 ▶); cell refinement: *CrysAlis PRO*; data reduction: *CrysAlis PRO*; program(s) used to solve structure: *SHELXS97* (Sheldrick, 2008 ▶); program(s) used to refine structure: *SHELXL97* (Sheldrick, 2008 ▶); molecular graphics: *ORTEP-3 for Windows* (Farrugia, 1997 ▶) and *PLATON* (Spek, 2009 ▶); software used to prepare material for publication: *WinGX* (Farrugia, 1999 ▶).

## Supplementary Material

Crystal structure: contains datablock(s) I, global. DOI: 10.1107/S1600536812000165/im2349sup1.cif

Structure factors: contains datablock(s) I. DOI: 10.1107/S1600536812000165/im2349Isup2.hkl

Supplementary material file. DOI: 10.1107/S1600536812000165/im2349Isup3.cml

Additional supplementary materials:  crystallographic information; 3D view; checkCIF report

## Figures and Tables

**Table 1:** Hydrogen-bond geometry (Å, °)

*D*—H⋯*A*	*D*—H	H⋯*A*	*D*⋯*A*	*D*—H⋯*A*
O3—H3⋯O2^i^	0.82	2.31	3.128 (2)	171
C8—H8*B*⋯O1^ii^	0.97	2.58	3.500 (2)	158
C7—H7⋯O1^iii^	0.98	2.65	3.579 (2)	159

Symmetry codes: (i) 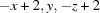; (ii) 
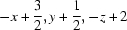
; (iii) 
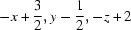
.

**Fig. 1.**
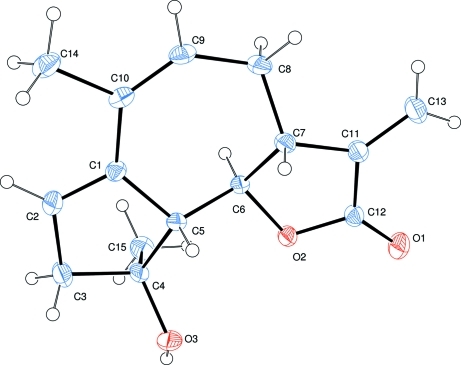
: Molecular structure of the title compound with displacement ellipsoids drawn at the 30% probability level. H atoms are represented as small spheres of arbitrary radii.

**Fig. 2.**
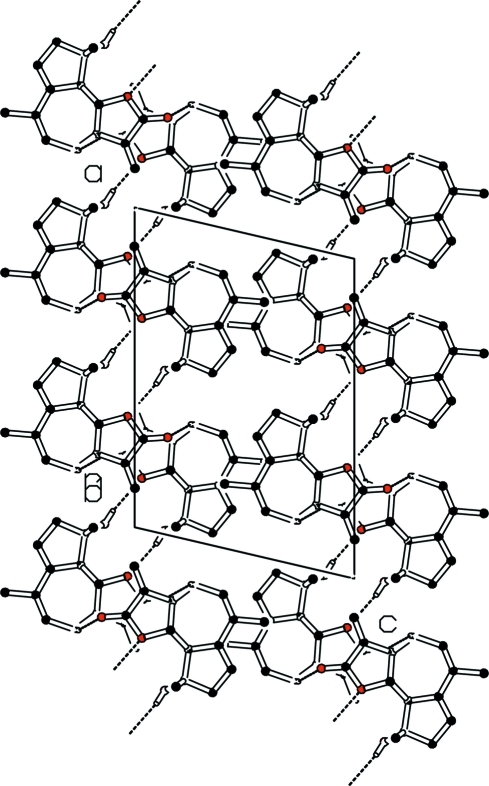
: Partial packing view showing the O–H···O hydrogen bonds as dashed lines. H atoms not involved in hydrogen bonding have been omitted for clarity.

## supplementary crystallographic information

### Comment

*Anvillea radiata* is a plant that grows in northern Africa and particularly found in the two Maghreb countries, Morocco and Algeria. This plant is used in traditional local medicine for the treatment of dysentery, gastric-intestinal disorders (Bellakhdar, 1997), hypoglycemic activity (Qureshi *et al.*, 1990), and has been reported to have antitumoral activity (Abdel Sattar *et al.*, 1996). In our study of different Moroccan endemic plants, we have demonstrated that the aerial parts of *Anvillea radiata* could be used as a renewable source of 9-hydroxyparthenolide (El Hassany, *et al.*, 2004). In order to prepare products with high added value that can be used in pharmacology and cosmetics industry, we studied the chemical reactivity of this major constituent of *Anvillea radiata*. Thus, treatment of this sesquiterpene with Bi(OTf)_3_ in dichloromethane (El Haib *et al.*, 2011) leads to the litle compound with a yield of 60%. The crystal structure of (I) is reported herein.

The molecule contains three fused rings which exhibit different conformations. The molecular structure of (I), Fig.1, shows the lactone ring to adopt a twisted conformation, as indicated by Cremer & Pople (1975) puckering parameters Q = 0.2747 (18) Å and φ = 59.0 (4)° while the other five-membered ring displays an envelope conformation with Q = 0.291 (2)Å and φ = 290.3 (4)°. The seven-membered ring has a chair conformation with QT = 0.5793 (16) Å, θ2 = 16.44 (20)°, φ2 = -139.46 (75)° and φ3 =107.00 (25). In the crystal structure, molecules are connected by O—H···O hydrogen bonds connecting molecules into dimers that again are interconnected by C—H···O interactions to produce infinite chains along *b* axis (Table 1).

### Experimental

Bi(OTf)_3 _(39 mg, 6 x 10^–2^ mmol) was added to a stirred solution of 9β- hydroxyparthenolide (600 mg, 2.27 mmol) in dichloromethane (10 ml). The reaction mixture is left stirring for three hours at room temperature. After completion of the reaction, a saturated solution of NaHCO_3_ was added and the resulting mixture is extracted three times (3 x 20 mL) with dichloromethane. The organic phases are combined and dried over Na_2_SO_4_ and evaporated under vacuum. Chromatography of the residue obtained on a column of silica gel eluting with hexane - ethyl acetate (85/15) allowed the isolation of the title compound (334 mg, 1.35 mmol) with a yield of 60%. Recrystallization from ethyl acetate at room temperature yielded single crystals of the title compound.

### Refinement

All H atoms were fixed geometrically and treated as riding with C—H = 0.96 Å (methyl), 0.97 Å (methylene), 0.98 Å (methine) with *U*_iso_(H) = 1.2*U*_eq_(methylene, methine) or *U*_iso_(H) = 1.5*U*_eq_(methyl, OH). In the absence of significant anomalous scattering, the absolute configuration could not be reliably determined and thus the Friedel pairs were merged and any references to the Flack parameter were removed.

### Crystal data

**Table:**

C_15_H_18_O_3_	*F*(000) = 528
*M**_r_* = 246.29	*D*_x_ = 1.307 Mg m^−^^3^
Monoclinic, *C*2	Mo *K*α radiation, λ = 0.71073 Å
Hall symbol: C 2y	Cell parameters from 9785 reflections
*a* = 15.6732 (9) Å	θ = 3–26.4°
*b* = 7.4208 (4) Å	µ = 0.09 mm^−^^1^
*c* = 11.0544 (6) Å	*T* = 180 K
β = 103.169 (6)°	Box, pale yellow
*V* = 1251.90 (12) Å^3^	0.42 × 0.19 × 0.12 mm
*Z* = 4	

### Data collection

**Table:**

Agilent Xcalibur Eos Gemini Ultra diffractometer	1378 independent reflections
Radiation source: fine-focus sealed tube	1313 reflections with *I* > 2σ(*I*)
graphite	*R*_int_ = 0.029
Detector resolution: 8.2632 pixels mm^-1^	θ_max_ = 26.4°, θ_min_ = 3.1°
ω scans	*h* = −19→19
Absorption correction: multi-scan (CrysAlis PRO; Agilent, 2010)	*k* = −9→9
*T*_min_ = 0.631, *T*_max_ = 1.000	*l* = −13→13
13492 measured reflections	

### Refinement

**Table:**

Refinement on *F*^2^	Primary atom site location: structure-invariant direct methods
Least-squares matrix: full	Secondary atom site location: difference Fourier map
*R*[*F*^2^ > 2σ(*F*^2^)] = 0.030	Hydrogen site location: inferred from neighbouring sites
*wR*(*F*^2^) = 0.080	H-atom parameters constrained
*S* = 1.05	*w* = 1/[σ^2^(*F*_o_^2^) + (0.0581*P*)^2^ + 0.1945*P*] where *P* = (*F*_o_^2^ + 2*F*_c_^2^)/3
1378 reflections	(Δ/σ)_max_ < 0.001
166 parameters	Δρ_max_ = 0.19 e Å^−^^3^
1 restraint	Δρ_min_ = −0.15 e Å^−^^3^

### Special details

**Table:**

Experimental. Empirical absorption correction using spherical harmonics, implemented in SCALE3 ABSPACK scaling algorithm. CrysAlisPro (Agilent Technologies, 2010)
Geometry. All s.u.'s (except the s.u. in the dihedral angle between two l.s. planes) are estimated using the full covariance matrix. The cell s.u.'s are taken into account individually in the estimation of s.u.'s in distances, angles and torsion angles; correlations between s.u.'s in cell parameters are only used when they are defined by crystal symmetry. An approximate (isotropic) treatment of cell s.u.'s is used for estimating s.u.'s involving l.s. planes.
Refinement. Refinement of *F*^2^ against ALL reflections. The weighted *R*-factor *wR* and goodness of fit *S* are based on *F*^2^, conventional *R*-factors *R* are based on *F*, with *F* set to zero for negative *F*^2^. The threshold expression of *F*^2^ > 2σ(*F*^2^) is used only for calculating *R*-factors(gt) *etc*. and is not relevant to the choice of reflections for refinement. *R*-factors based on *F*^2^ are statistically about twice as large as those based on *F*, and *R*- factors based on ALL data will be even larger.

### Fractional atomic coordinates and isotropic or equivalent isotropic displacement parameters (Å^2^)

**Table:**

	*x*	*y*	*z*	*U*_iso_*/*U*_eq_	
C1	0.80721 (11)	0.4633 (2)	0.61618 (14)	0.0267 (3)	
C2	0.87339 (12)	0.4573 (3)	0.55907 (15)	0.0380 (4)	
H2	0.8666	0.4746	0.4741	0.046*	
C3	0.95947 (12)	0.4201 (4)	0.64549 (17)	0.0455 (5)	
H3A	1.0049	0.4976	0.6278	0.055*	
H3B	0.9766	0.2953	0.6399	0.055*	
C4	0.94229 (10)	0.4614 (3)	0.77340 (14)	0.0322 (4)	
C5	0.84280 (10)	0.4259 (2)	0.75363 (14)	0.0246 (3)	
H5	0.8353	0.2968	0.7666	0.030*	
C6	0.79591 (9)	0.5247 (2)	0.83742 (14)	0.0233 (3)	
H6	0.7950	0.6540	0.8192	0.028*	
C7	0.70300 (10)	0.4572 (2)	0.82786 (14)	0.0265 (3)	
H7	0.7016	0.3276	0.8106	0.032*	
C8	0.63569 (11)	0.5483 (3)	0.72710 (16)	0.0338 (4)	
H8A	0.5782	0.5047	0.7319	0.041*	
H8B	0.6372	0.6766	0.7443	0.041*	
C9	0.64585 (11)	0.5222 (3)	0.59733 (16)	0.0323 (4)	
H9	0.5935	0.5305	0.5378	0.039*	
C10	0.71551 (11)	0.4891 (2)	0.54969 (14)	0.0287 (4)	
C11	0.69280 (10)	0.4841 (2)	0.95746 (15)	0.0283 (3)	
C12	0.78211 (11)	0.4836 (2)	1.03860 (15)	0.0290 (3)	
C13	0.62238 (13)	0.5135 (3)	1.00137 (19)	0.0401 (5)	
H13A	0.6280	0.5349	1.0857	0.048*	
H13B	0.5672	0.5129	0.9479	0.048*	
C14	0.70133 (13)	0.4837 (3)	0.41040 (15)	0.0402 (4)	
H14A	0.6403	0.5011	0.3734	0.060*	
H14B	0.7198	0.3690	0.3855	0.060*	
H14C	0.7348	0.5777	0.3835	0.060*	
C15	0.96829 (12)	0.6516 (3)	0.81170 (19)	0.0416 (5)	
H15A	0.9542	0.6767	0.8901	0.062*	
H15B	0.9371	0.7335	0.7501	0.062*	
H15C	1.0302	0.6660	0.8194	0.062*	
O1	0.80505 (9)	0.4767 (2)	1.14968 (11)	0.0415 (3)	
O2	0.84070 (7)	0.49325 (18)	0.96669 (9)	0.0290 (3)	
O3	0.98840 (8)	0.3361 (2)	0.86299 (13)	0.0437 (4)	
H3	1.0346	0.3813	0.9005	0.065*	

### Atomic displacement parameters (Å^2^)

**Table:**

	*U*^11^	*U*^22^	*U*^33^	*U*^12^	*U*^13^	*U*^23^
C1	0.0301 (8)	0.0277 (8)	0.0211 (7)	0.0032 (7)	0.0037 (6)	−0.0003 (7)
C2	0.0369 (9)	0.0543 (12)	0.0231 (7)	0.0066 (10)	0.0078 (7)	0.0013 (9)
C3	0.0317 (9)	0.0765 (16)	0.0308 (9)	0.0111 (10)	0.0126 (7)	0.0002 (9)
C4	0.0201 (7)	0.0513 (11)	0.0253 (7)	0.0084 (8)	0.0054 (6)	0.0037 (8)
C5	0.0222 (7)	0.0281 (8)	0.0230 (7)	0.0048 (6)	0.0039 (5)	0.0012 (6)
C6	0.0211 (7)	0.0267 (8)	0.0215 (7)	0.0020 (6)	0.0038 (5)	0.0009 (6)
C7	0.0219 (7)	0.0281 (8)	0.0299 (8)	0.0012 (6)	0.0066 (6)	0.0011 (7)
C8	0.0204 (7)	0.0410 (10)	0.0386 (10)	0.0043 (7)	0.0037 (7)	0.0056 (8)
C9	0.0241 (8)	0.0351 (10)	0.0325 (8)	−0.0005 (7)	−0.0044 (6)	0.0052 (7)
C10	0.0316 (8)	0.0247 (8)	0.0258 (7)	0.0001 (7)	−0.0018 (6)	0.0015 (7)
C11	0.0298 (8)	0.0238 (8)	0.0337 (8)	0.0013 (7)	0.0122 (6)	0.0021 (7)
C12	0.0336 (8)	0.0276 (8)	0.0282 (8)	0.0031 (8)	0.0122 (6)	−0.0002 (7)
C13	0.0361 (9)	0.0428 (11)	0.0471 (10)	0.0010 (8)	0.0218 (8)	0.0011 (9)
C14	0.0467 (10)	0.0422 (11)	0.0261 (8)	0.0043 (10)	−0.0030 (7)	0.0002 (8)
C15	0.0245 (8)	0.0556 (12)	0.0437 (10)	−0.0066 (8)	0.0054 (7)	0.0022 (10)
O1	0.0481 (7)	0.0523 (8)	0.0255 (6)	0.0011 (7)	0.0114 (5)	−0.0005 (6)
O2	0.0244 (5)	0.0407 (7)	0.0218 (5)	0.0026 (5)	0.0049 (4)	−0.0002 (5)
O3	0.0232 (6)	0.0660 (10)	0.0394 (7)	0.0125 (6)	0.0021 (5)	0.0152 (7)

### Geometric parameters (Å, °)

**Table:**

C1—C2	1.332 (2)	C8—C9	1.492 (3)
C1—C10	1.470 (2)	C8—H8A	0.9700
C1—C5	1.520 (2)	C8—H8B	0.9700
C2—C3	1.491 (2)	C9—C10	1.339 (3)
C2—H2	0.9300	C9—H9	0.9300
C3—C4	1.530 (2)	C10—C14	1.505 (2)
C3—H3A	0.9700	C11—C13	1.321 (2)
C3—H3B	0.9700	C11—C12	1.481 (2)
C4—O3	1.429 (2)	C12—O1	1.199 (2)
C4—C15	1.503 (3)	C12—O2	1.3464 (19)
C4—C5	1.547 (2)	C13—H13A	0.9300
C5—C6	1.498 (2)	C13—H13B	0.9300
C5—H5	0.9800	C14—H14A	0.9600
C6—O2	1.4602 (18)	C14—H14B	0.9600
C6—C7	1.520 (2)	C14—H14C	0.9600
C6—H6	0.9800	C15—H15A	0.9600
C7—C11	1.491 (2)	C15—H15B	0.9600
C7—C8	1.508 (2)	C15—H15C	0.9600
C7—H7	0.9800	O3—H3	0.8200
			
C2—C1—C10	123.05 (14)	C9—C8—C7	116.23 (15)
C2—C1—C5	108.64 (14)	C9—C8—H8A	108.2
C10—C1—C5	128.16 (14)	C7—C8—H8A	108.2
C1—C2—C3	113.02 (14)	C9—C8—H8B	108.2
C1—C2—H2	123.5	C7—C8—H8B	108.2
C3—C2—H2	123.5	H8A—C8—H8B	107.4
C2—C3—C4	103.37 (14)	C10—C9—C8	132.66 (15)
C2—C3—H3A	111.1	C10—C9—H9	113.7
C4—C3—H3A	111.1	C8—C9—H9	113.7
C2—C3—H3B	111.1	C9—C10—C1	128.26 (14)
C4—C3—H3B	111.1	C9—C10—C14	117.63 (15)
H3A—C3—H3B	109.1	C1—C10—C14	114.06 (15)
O3—C4—C15	110.74 (15)	C13—C11—C12	122.10 (16)
O3—C4—C3	110.03 (16)	C13—C11—C7	131.05 (16)
C15—C4—C3	110.80 (17)	C12—C11—C7	106.76 (13)
O3—C4—C5	108.76 (15)	O1—C12—O2	121.41 (15)
C15—C4—C5	113.55 (15)	O1—C12—C11	129.93 (15)
C3—C4—C5	102.67 (13)	O2—C12—C11	108.66 (13)
C6—C5—C1	114.13 (13)	C11—C13—H13A	120.0
C6—C5—C4	116.75 (13)	C11—C13—H13B	120.0
C1—C5—C4	103.81 (12)	H13A—C13—H13B	120.0
C6—C5—H5	107.2	C10—C14—H14A	109.5
C1—C5—H5	107.2	C10—C14—H14B	109.5
C4—C5—H5	107.2	H14A—C14—H14B	109.5
O2—C6—C5	109.43 (12)	C10—C14—H14C	109.5
O2—C6—C7	104.76 (11)	H14A—C14—H14C	109.5
C5—C6—C7	113.14 (13)	H14B—C14—H14C	109.5
O2—C6—H6	109.8	C4—C15—H15A	109.5
C5—C6—H6	109.8	C4—C15—H15B	109.5
C7—C6—H6	109.8	H15A—C15—H15B	109.5
C11—C7—C8	116.08 (14)	C4—C15—H15C	109.5
C11—C7—C6	101.53 (12)	H15A—C15—H15C	109.5
C8—C7—C6	113.67 (14)	H15B—C15—H15C	109.5
C11—C7—H7	108.4	C12—O2—C6	110.23 (12)
C8—C7—H7	108.4	C4—O3—H3	109.5
C6—C7—H7	108.4		

### Hydrogen-bond geometry (Å, °)

**Table:**

*D*—H···*A*	*D*—H	H···*A*	*D*···*A*	*D*—H···*A*
O3—H3···O2^i^	0.82	2.31	3.128 (2)	171
C8—H8B···O1^ii^	0.97	2.58	3.500 (2)	158
C7—H7···O1^iii^	0.98	2.65	3.579 (2)	159

Symmetry codes: (i) −*x*+2, *y*, −*z*+2; (ii) −*x*+3/2, *y*+1/2, −*z*+2; (iii) −*x*+3/2, *y*−1/2, −*z*+2.
